# *Frankliniella species* from China, with nomenclatural changes and illustrated key (Thysanoptera, Thripidae)

**DOI:** 10.3897/zookeys.873.36863

**Published:** 2019-08-29

**Authors:** Zhaohong Wang, Laurence Mound, Xiaoli Tong

**Affiliations:** 1 Department of Entomology, College of Agriculture, South China Agricultural University, Guangzhou 510642, China Australian National Insect Collection Canberra Australia; 2 Australian National Insect Collection, CSIRO, Canberra, Australia South China Agricultural University Guangzhou China

**Keywords:** distribution, identification, new synonyms

## Abstract

Species of the genus *Frankliniella* are almost all originally from the New World. Although eleven species in this genus have been listed from China before, only seven species here are recognised. The records of *F. pallida* and *F. tritici* from China are rejected as inadequately supported, *F. hainanensis* is a new synonym of *F. schultzei*, and *F. zizaniophila* is now considered a member of the genus *Iridothrips*. An identification key for the seven species is provided here, two of them are widespread across the Palearctic (*F. intonsa* and *F. tenuicornis*), one is presumably Oriental (*F. lilivora*), and four are introduced from the Americas (*F. occidentalis*, *F. schultzei*, *F. cephalica* and *F. williamsi*).

## Introduction

The genus *Frankliniella* is one of the most species-rich genera of Thysanoptera, with 238 species listed currently (ThripsWiki 2019). Most of these are from Central and South America (Mound and Marullo 1996), with only six from the Old World. Although some species in the genus appear to be host specific on particular plants, a surprisingly large number of *Frankliniella* are polyphagous, breeding in the flowers and leaves of a range of different plants. Species with this type of biology are effectively pre-adapted to become crop pests.

In China, the Western Flower Thrips, *Frankliniella occidentalis*, is currently one of the most important insect pests of agriculture and horticulture (Cao et al. 2019), both for its direct feeding damage and for its ability to transmit tospoviruses to plants (Reitz et al. 2011). Originally from western USA, this pest is now established widely around the world, presumably distributed by the horticultural trade (Kirk and Terry 2003). A recent list of Thysanoptera species recorded from China included 12 species of *Frankliniella* (Mirab-balou et al. 2011), but this was reduced recently to 11 with some further records considered doubtful (Zhang et al. 2018). Several of the records are here recognised as incorrect, and the number of species in this genus that are validly recorded from China is now considered to be no more than seven. Two of these seven are clearly native to Palearctic, *F. intonsa* and *F. tenuicornis*, and these are often abundant with a natural distribution across China. One rare species, *F. lilivora*, was described on specimens taken in quarantine in Japan and apparently imported from China (Kurosawa 1937), but it is not known from any specimens collected in China. The remaining four species are all clearly introduced to China from other parts of the world, *F. occidentalis* from western north America, *F. cephalica* and *F. williamsi* from Meso-America, and *F. schultzei* from South America (or possibly from Africa).

## Unverified species records from China

As discussed below under *F. schultzei*, one species described from Hainan as *F. hainanensis* is here recognised as a synonym of that widespread tropical pest species. A further difference from the checklist provided by Mirab-balou et al. (2011) is that the species described from China as *F. zizaniophila* is now considered a species of *Iridothrips* (Wang et al. 2019). Two further problems with the published checklist are as follows. *F. pallida* (Uzel) was recorded from China (Feng 1992) based on two males collected from flowers of *Paeonia* in Yangling, Shaanxi Province. The author provided no indication as to how these specimens were identified. Moreover, the two specimens were not distinguished from the very similar males of the two common species, *intonsa* and *occidentalis*. In view of the lack of information, the record of *pallida* from China is considered unsubstantiated. *F. tritici* is listed from China based on a single female taken in Taiwan. This female was originally described by Moulton (1948) as *F. salicis* but was subsequently recognised by Nakahara (1997: 377) as a female of *tritici*. No other specimens of *tritici* have ever been reported from Taiwan (Wang 2002), nor from mainland China, nor yet from anywhere outside of the Americas (Cavalleri and Mound 2012). In the absence of any further records, it is here assumed that the female of *salicis* came from somewhere in North America and was mislabeled during slide mounting. A similar situation was reported (Marullo and Mound 1994) concerning the record of a Californian species from India by Moulton (1927).

## Materials and methods

Nomenclatural details for all taxa mentioned in this paper are available in ThripsWiki (2019). Examined specimens were slide-mounted in Canada balsam using the method of Zhang et al. (2006), and specimens are deposited in **SCAU** (Insect Collection, South China Agricultural University, Guangzhou) and **ANIC** (Australian National Insect Collection, CSIRO, Canberra). Observations were made with a Nikon Eclipse 80i phase contrast microscope, and the illustrations taken through a Leica DM 2500 microscope with DIC illumination using Automontage software, although Figure 18 was provided by Masami Masumoto.

## Key to *Frankliniella* species in China

**Table:** 

1	Abdominal tergite VIII with complete posteromarginal comb of long slender microtrichia, sometimes arising from broad triangular bases (Figs 12, 14, 16)	**2**
–	Abdominal tergite VIII posteromarginal comb absent at least medially, some with irregular weak teeth laterally (Figs 13, 15, 17)	**4**
2	Postocular setae pair IV short, much shorter than distance between hind ocelli (Fig. 4); pronotum anterior margin generally with 1 pair of small setae between the major anteromarginal pair (Fig. 7)	*** intonsa ***
–	Postocular setae pair IV as long as or longer than distance between hind ocelli (Fig. 5); pronotum anterior margin generally with 2 pairs of small setae between major anteromarginal pair (Fig. 3)	**3**
3	Head and body pale yellow, tergites uniformly pale; sternite II usually with 1 or 2 long discal setae medially (Fig. 8)	*** williamsi ***
–	Body colour variable brown to yellow, if tergites pale then with dark area medially (Fig. 11); sternite II without discal setae medially	*** occidentalis ***
4	Antennal segment II with pair of stout dark setae arising from apical projection; segment III pedicel with sharp-edged ring, base of segment cup-shaped (Fig. 10)	*** cephalica ***
–	Antennal segment II without a pair of stout dorsal apical setae; segment III pedicel without prominent ring (Fig. 9)	**5**
5	Ocellar setae pair III arising within ocellar triangle, between hind ocelli (Fig. 6)	*** schultzei ***
–	Ocellar setae pair III arising further apart, near margins of ocellar triangle (Fig. 2)	**6**
6	Mesonotum with median pair of setae near posterior margin; metanotum with median pair of setae at anterior margin, campaniform sensilla absent (Fig. 19)	*** tenuicornis ***
–	Mesonotum with median pair of setae far from posterior margin (Fig. 18); metanotum with median pair of setae behind anterior margin, campaniform sensilla present	*** lilivora ***

**Figures 1–11.**
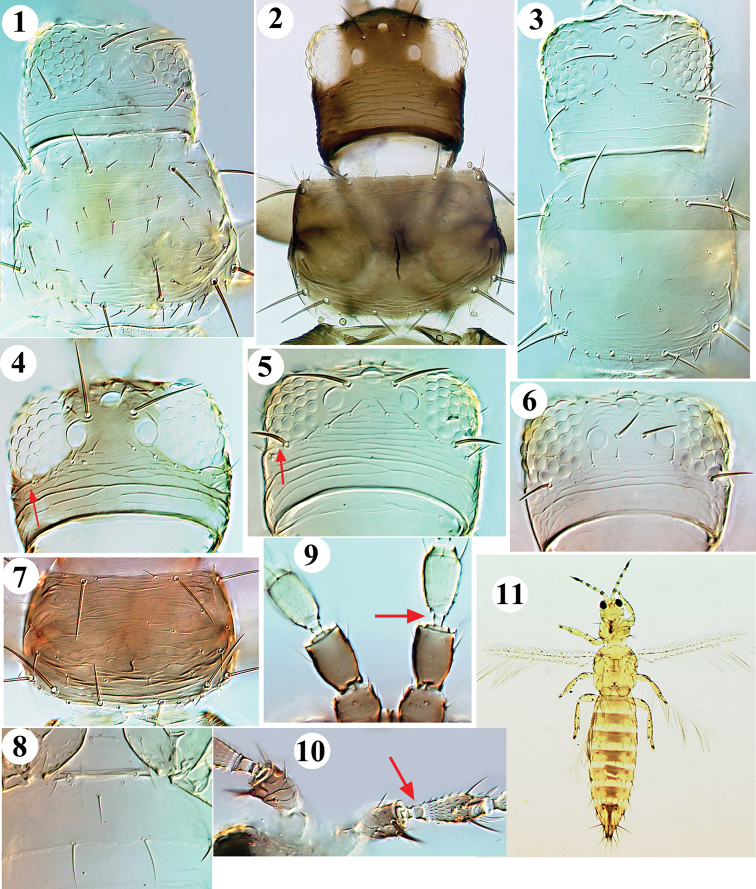
*Frankliniella* from China. Head & Pronotum: **1***cephalica***2***tenuicornis***3***williamsi*. Head: **4***intonsa***5***occidentalis***6***schultzei*. **7***intonsa* pronotum. **8***williamsi* sternite II. Antennal segments II–III: **9***intonsa***10***cephalica***11***occidentalis* female.

**Figures 12–19.**
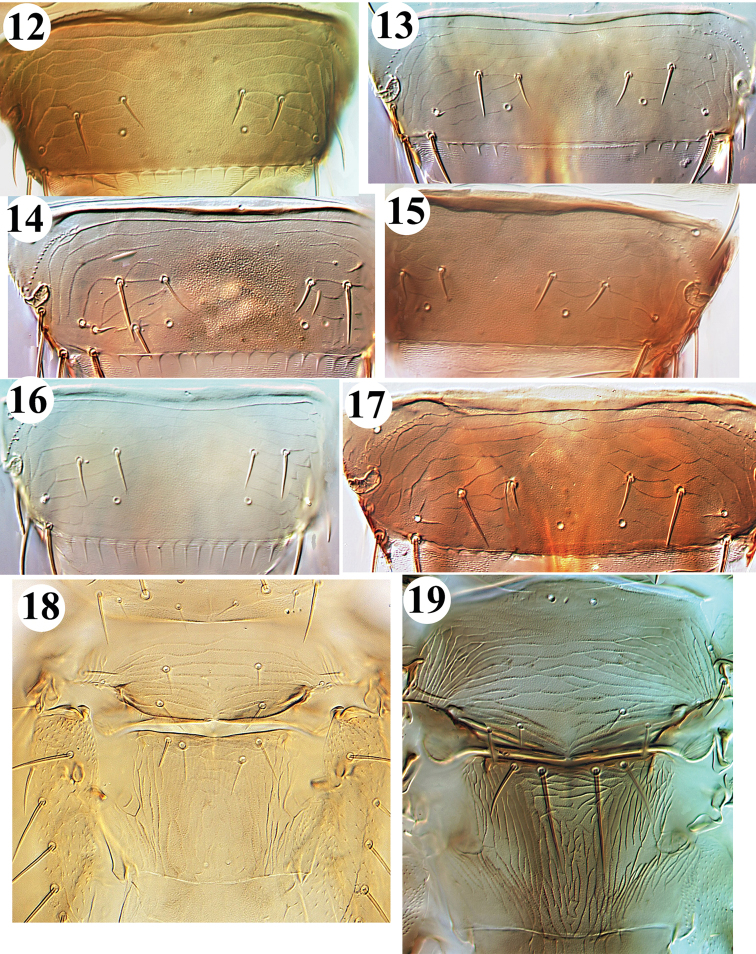
*Frankliniella* from China. Tergite VIII: **12***intonsa***13***cephalica***14***occidentalis***15***schultzei***16***williamsi***17***tenuicornis*. Meso- & metanotum: **18***lilivora***19***tenuicornis*.

### *Frankliniella cephalica* (Crawford)

**Remarks.** Native to Central America and the Caribbean where it is one of the most common flower-thrips, this species has been studied in tropical areas in China from Guangdong, Guangxi, Hainan as well as Taiwan (Wang et al. 2010; Tong and Lv 2013). It has also been seen from Okinawa, Japan (Masumoto and Okajima 2004). Structurally it is unusual in that the base of the third antennal segment is strongly expanded into a sharp-edged ring (Fig. 10). In Central America this species is common in white flowers (Mound and Marullo 1996), but in China and Japan it seems to be particularly associated with the flowers of *Bidens pilosa* [Asteraceae] (Masumoto and Okajima 2004; Tong and Lv 2013). *F. cephalica* was reported as a tospovirus vector (Ohnishi et al. 2006), and the plant *B. pilosa* has been reported as a host of Tomato spotted wilt virus in China (Huang et al. 2016). Because of its vector ability, there is a high possibility of *F. cephalica* becoming a crop pest in China.

### *Frankliniella intonsa* (Trybom)

**Remarks.** This polyphagous flower thrips is widespread in the Old World and is also reported from northwestern America (Mound et al. 2019). It is one of the most common species in China, being widespread from Xinjiang to Taiwan, feeding and breeding in many different flowers. This species has been recorded as a tospovirus vector (Wijkamp et al. 1995), but its transmission efficiency was relatively low compared to *F. occidentalis* (Sakurai et al. 2004). It has been considered an important pest in China, Japan and Korea, involving hundreds of experiments but with limited evidence of yield reduction on crops. In Taiwan, this species was considered important in the international flower trade due to quarantine problems (Wang 2002). From our collection experience in the field, this species is possibly a significant pollinator.

### *Frankliniella lilivora* Kurosawa

**Remarks.** Described from Japan on specimens taken in quarantine on lily bulbs imported from China (Dalian and Shanghai) and Korea (Kurosawa 1937). There are no records of the species from China, including Taiwan, and only a few specimens are known from Japan. According to the original description, it is different in having short antennae and no posteromarginal comb but a few weak and irregular teeth laterally on tergite VIII. However, it is readily distinguished from other *Frankliniella* species by the anterior position of the mesonotal median setae, and the position of the metanotal median setae behind the anterior margin (Fig. 18).

### *Frankliniella occidentalis* (Pergande)

**Remarks.** Originally from the western parts of North America, it is now widespread across temperate parts of the world (Kirk and Terry 2003). It causes extensive damage to many horticultural crops, both through direct feeding damage particularly in young buds, and also through vectoring tospoviruses, it has been referred to as the most studied thrips species (Reitz 2009). Although normally phytophagous, *occidentalis* is also known to function as a predator of spider mites on leaves (Trichilo and Leigh 1986). In China, it was first recorded as a major thrips pest of horticulture and ornamental plant production in glasshouses in Beijing (Zhang et al. 2003). The species has now been found in many provinces in China (Yang et al. 2012), mainly in provinces involved in the extensive flower trade (Lv et al. 2011), and a wide potential distribution across China was reported (Cheng et al. 2006). It is not usually found in lowland tropical areas with a high humidity, but it can live on plants grown in tropical montane areas. This species exhibits variation in body colour, with dark, light, and intermediate colour morphs reported (Bryan and Smith 1956). The dark forms can be particularly common in cooler montane areas and in winter in Yunnan (Shen et al. 2015), while populations associated with crops usually involve just the intermediate form (Fig. 11). A further problem with this species is that cryptic species have been found using molecular data, but these species lack supporting biological or morphological evidence (Rugman-Jones et al. 2010).

### *Frankliniella schultzei* (Trybom)

**Remarks.** Commonly known as the Tomato Thrips, this species has probably been transported by human trade in plants for many years. As a result, its country of origin remains unclear, and could have been either South America or Africa. A species from China, *F. hainanensis* (Zheng et al. 2009) might be a synonym of *schultzei* according to its original descriptions and illustrations, which was also indicated by Zhang et al. (2018). Recently, Shimeng Zhang helped examine the type specimens of *hainanensis* and confirmed that it is a new synonym of *schultzei*. A further species described from Taiwan, *F. gossypii* (Shiraki 1912), was recognised as a synonym of *schultzei* by Nakahara (1997). This tropical species is exceptional within the genus in having ocellar setae pair III arising close together within the ocellar triangle (Fig. 6) and tergite VIII with almost no posteromarginal comb (Fig. 15). As an important crop pest and vector of several tospovirus diseases (Riley et al. 2011), *schultzei* shares with *occidentalis* the remarkable ability to act as a predator of leaf mites (Wilson et al. 1996). The species is widespread in tropical and subtropical countries around the world, and has been found in southern China in Guangxi, Guangdong, Hainan, Fujian and Taiwan (Zheng et al. 2009; Wang et al. 2010; Xie et al. 2011), but molecular studies in Australia have suggested that a series of sibling species may be involved (Hereward et al. 2017).

### *Frankliniella tenuicornis* (Uzel)

**Remarks.** This is one of the few species of *Frankliniella* that breeds on grasses, including cereal crops, and is one of the main pests that feed on young leaves of *Zea mays* in northern China (Han et al. 1979; Zhang et al. 2014a, b). It occurs widely across the Holarctic (Mound et al. 2018), and in China is found mainly in the Palaearctic areas, based on our collecting experiences (and communication with Hongrui Zhang). Records from southern provinces of China (Han 1997) require further confirmation. As a large, dark brown species, it is distinctive in the genus for the projection of the head in front of the compound eyes (Fig. 2). Moreover, postocular setae pair IV are small, and the pronotal posteromarginal setae IV are longer than pairs III and V.

### *Frankliniella williamsi* Hood

**Remarks.** This yellow species is closely associated with crops of *Zea mays*, on which it is reported to be a virus vector in addition to causing feeding damage to young leaves (O’Donnell and Mound 2016). It presumably originated in Central or South America in association with maize, although it has also been taken from other species of Poaceae. It is widespread in tropical areas around the world, and in China is reported from Hainan and Taiwan. In most parts of the world it is easy to recognise from other pale coloured *Frankliniella* species by the presence of one or two discal setae on the second abdominal sternite (Fig. 8).
